# Individual differences in emotion-cognition interactions: emotional valence interacts with serotonin transporter genotype to influence brain systems involved in emotional reactivity and cognitive control

**DOI:** 10.3389/fnhum.2013.00327

**Published:** 2013-07-04

**Authors:** Melanie Stollstorff, Yuko Munakata, Arielle P. C. Jensen, Ryan M. Guild, Harry R. Smolker, Joseph M. Devaney, Marie T. Banich

**Affiliations:** ^1^Institute of Cognitive Science, University of Colorado BoulderBoulder, CO, USA; ^2^Department of Psychology and Neuroscience, University of Colorado BoulderBoulder, CO, USA; ^3^Department of Integrative Systems Biology, Research Center for Genetic Medicine, Children's National Medical CenterWashington, DC, USA

**Keywords:** 5-HTTLPR, Stroop, fMRI, prefrontal cortex (PFC), eye-gaze, anxiety, positive affect

## Abstract

The serotonin transporter gene (5-HTTLPR) influences emotional reactivity and attentional bias toward or away from emotional stimuli, and has been implicated in psychopathological states, such as depression and anxiety disorder. The short allele is associated with increased reactivity and attention toward *negatively-valenced* emotional information, whereas the long allele is associated with increased reactivity and attention toward *positively-valenced* emotional information. The neural basis for individual differences in the ability to exert cognitive control over these bottom-up biases in emotional reactivity and attention is unknown, an issue investigated in the present study. Healthy adult participants were divided into two groups, either homozygous carriers of the 5-HTTLPR long allele or homozygous carriers of the short allele, and underwent functional magnetic resonance imaging (fMRI) while completing an Emotional Stroop-like task that varied in the congruency of task-relevant and task-irrelevant information and the emotional valence of the task-irrelevant information. Behaviorally, participants demonstrated the classic “Stroop effect” (responses were slower for incongruent than congruent trials), which did not differ by 5-HTTLPR genotype. However, fMRI results revealed that genotype influenced the degree to which neural systems were engaged depending on the valence of the conflicting task-irrelevant information. While the “Long” group recruited prefrontal control regions and superior temporal sulcus during conflict when the task-irrelevant information was positively-valenced, the “Short” group recruited these regions during conflict when the task-irrelevant information was negatively-valenced. Thus, participants successfully engaged cognitive control to overcome conflict in an emotional context using similar neural circuitry, but the engagement of this circuitry depended on emotional valence and 5-HTTLPR status. These results suggest that the interplay between emotion and cognition is modulated, in part, by a genetic polymorphism that influences serotonin neurotransmission.

## Introduction

How does emotion influence cognition? Here we examine the degree to which cognitive control, the ability to engage in goal-directed behavior, is influenced by salient but task-irrelevant information that is emotional in nature. Currently, the evidence is divided, with some studies suggesting that emotional information can facilitate, impede, or have no effect on cognitive control (Cohen and Henik, 2012). Research has identified factors that can influence or mediate these effects, including the valence of the emotional material (i.e., positive vs. negative e.g., Kahan and Hely, 2008), individual differences in negative affect such as anxiety (Cisler and Wolitzky-Taylor, 2011), and genetic polymorphisms that may contribute to these individual differences, such as the serotonin transporter gene (Beevers and Wells, 2009). The present study aims to investigate the interaction of these factors in healthy individuals and in doing so, shed light on the underlying neurobiology of emotion-cognition interactions.

One of the most replicated findings regarding genetic polymorphisms is that the 5-HTTLPR genotype influences emotional reactivity to negative information (Pergamin-Hight et al., 2012) and sensitivity to stressors (Karg et al., 2011). A polymorphism in the promoter region of the serotonin transporter gene (5-HTTLPR) results in short (S) and long (L) variants. The S allele is linked to lower expression of serotonin transporter mRNA. Further, the L allele contains an A to G single nucleotide polymorphism (SNP rs25531) that influences transcriptional efficiency, rendering the L_G_ allele functionally similar to the S allele (Hu et al., 2006). A variety of evidence drawn from studies comparing S carriers (SS alone or with SL_G_) with homozygous L carriers (e.g., LL or L_A_L_A_) suggests that the S allele is associated with higher negative affect. First, genetic association studies suggest that the S allele contributes to risk for affective psychiatric disorders as it is overtransmitted in those patients (Caspi et al., 2003; Karg et al., 2011; but see Munafò et al., 2009). Second, healthy carriers of the S allele score higher on measures of depressive and anxiety-related behaviors (Lesch et al., 1996; Gonda et al., 2009; Lonsdorf et al., 2009). Third, they tend to show a stronger bias toward negative content (e.g., angry faces) in an emotional dot-probe task (Beevers and Wells, 2009; Pérez-Edgar et al., 2010) and show increased interference from negative stimuli (e.g., threat words or angry faces) in Stroop-like tasks (Koizumi et al., 2010). Fourth, numerous functional neuroimaging studies demonstrate that the amygdala, a critical brain region underlying emotional behavior, is more responsive to negative stimuli in healthy S carriers [see meta-analyses (Munafò et al., 2008; Murphy et al., 2013)]. Recent studies suggest that the Long allele may be associated with a bias away from negative stimuli and/or increased sensitivity to *positive* emotional stimuli. For example, L carriers show a bias away from negative stimuli (Kwang and Wells, 2010) and toward happy faces (Pérez-Edgar et al., 2010) in a behavioral dot-probe paradigm. Together, these findings indicate that S (and L_G_) carriers differ in emotional reactivity from L carriers (and L_A_ alone), with S carriers showing a “negativity bias” and L carriers potentially showing a “positivity bias.”

What is not clear is how such individual differences in emotional biases may interact with or influence the ability to exert cognitive control, a question we address here. However, there is good reason to believe that emotional biases are likely to influence the degree to which cognitive control can be exerted and the activation of neural systems supporting such control. For example, in non-clinical samples of individuals who do not reach criteria for a psychiatric disorder, a higher tendency toward anhedonic depression is associated with decreased activity in posterior regions of the dorsolateral prefrontal cortex during performance of a color-word Stroop task (Herrington et al., 2010). As the color-word Stroop task does not involve emotional information, but cognitive conflict, this finding suggests that individual differences in emotional biases may influence the activity of brain regions involved in cognitive control. Additional evidence suggests that engagement of cognitive control regions may be influenced not only by such trait individual differences, but also by the nature of task-irrelevant emotional information. For example, individuals high in anxious apprehension (i.e., worry) show greater activity in left lateral prefrontal regions in the face of emotionally negative as compared to neutral task-irrelevant words in an emotion-word Stroop task (Engels et al., 2007). As these two examples illustrate, both the emotional make-up of an individual as well as the emotional valence of task-irrelevant information may serve to influence neural systems that exert cognitive control.

In consideration of these prior findings, we investigated the effect of certain variants of the 5-HTTLPR genotype on neural systems underlying cognitive control. In prior studies of cognitive control examining individual differences in trait emotional biases, there have been two types of task-irrelevant information. In some cases, the task-irrelevant information has been emotional in nature (e.g., a task-irrelevant emotion word when the task goal is to identify the word's ink color). In these paradigms, cognitive control must be exerted in the face of such emotional information because it is likely to capture attention (Ishai et al., 2004). In other cases, cognitive control must be exerted because the non-emotional task-irrelevant information (e.g., a color word) conflicts, semantically and/or with regards to response-mappings, with the task-relevant information (e.g., the word's ink color, as in the case of the word “red” printed in blue ink) (see Banich et al., 2009 for a longer discussion).

In the present investigation, we utilize a task that allowed us to integrate these two types of task-irrelevant information to determine how genotype affects cognitive control. In our task (similar to that of Barnes et al., 2007), individuals were asked to press a button corresponding to a word (left, right) placed on the forehead of a face. On incongruent trials, the position of the person's pupils was opposite that of the word on the forehead (e.g., pupils on the left when the word says “right) and required more cognitive control than on congruent trials, in which the position of the person's pupils corresponds to the word on the forehead (e.g., pupils on the left when the word says “left”). Here cognitive control is required both because of the spatial incompatibility between the word and eye gaze, and also because eye gaze is a salient emotional feature of the face that will capture attention (Barnes et al., 2007; Schwartz et al., 2010; Vaidya et al., 2011).

In addition, we varied the emotional expression of the face to be negative, neutral or positive. Like the word in the standard emotion-word Stroop task, the facial expression in this task is unrelated to the task goals (which in the current task is to determine the spatial meaning of a word). Yet we can explore whether such information influences the ability to exert cognitive control. The emotional expression is likely to be a potent distractor as it, like eye gaze, is an integral part of the facial expression, which will attract attention.

We predicted that across all participants, the task should engage regions previously identified as underlying cognitive control and interference resolution, such as the dorsolateral prefrontal cortex (PFC), anterior cingulate cortex, and inferior frontal regions. In addition, it should also engage regions involved in face processing, most likely including the portions of the fusiform gyrus (Kanwisher and Yovel, 2006) and the superior temporal sulcus (STS), which has been found to be sensitive to aspects of facial expression that can change over time and have social significance, including eye gaze (Nummenmaa et al., 2010).

Our key prediction was that because of increased sensitivity to negative affective stimuli in S (and L_G_) carriers, carriers of the 5-HTTLPR S or L_G_ alleles (SS, SL_G_, L_G_L_G_; “Short”) would show differential activation of cognitive control systems during conflict when the emotional context was negative in nature. This prediction was based on the idea that the task-irrelevant negative information contained in the facial expression is likely to capture attention in these individuals, and make the implementation of cognitive control more demanding. We also predicted that this pattern should be absent or perhaps even reversed in the homozygous carriers of the L_A_ allele (L_A_L_A_; “Long”), who are likely to ignore negative information and/or be more sensitive to positive information. Our study did not include S/L_A_ heterozygotes because unlike the short and long carriers, it is not clear what bias they would show toward affective stimuli.

In conjunction, we also examined whether the two groups would differ in regards to the engagement of cognitive control regions in response to conflict that is not highly emotional in nature. There is at least some evidence that cognitive control mechanisms may differ between the groups (Fallgatter et al., 2004; Althaus et al., 2009; Holmes et al., 2010). To address this issue, we examined activation of these cognitive control and face-processing regions in a neutral emotion condition.

## Methods

### Participants

fMRI participants were drawn from a pool of 221 University of Colorado Boulder undergraduate students (105 male; 47.5%) of primarily European descent (93%) without history of psychiatric diagnosis or medication, who were right-handed and were native English speakers or fluent by age 10, who participated in the initial screen for course-credit or payment. Consent was acquired according to Institutional Review Board guidelines. Potential participants provided a saliva sample that was analyzed for 5-HTTLPR and the rs25531 SNP in the serotonin transporter gene (SLC6A4). Genotype frequencies were in Hardy-Weinberg equilibrium (*X*^2^ = 1.310, *df* = 2, *p* > 0.1). In light of evidence indicating functional similarity between the low-expressing S and L_G_ alleles (Hu et al., 2006), we included L_G_ carriers in the S group as done in past work (Armbruster et al., 2009). Carriers who had two copies of either the high-expressing (L_A_) or low-expressing (S or L_G_) alleles were invited to participate in the fMRI study. SL_A_ and L_A_L_G_ heterozygotes, that is, carriers of both high and low expressing alleles, were excluded in order to maximize observed allelic differences (Roiser et al., 2009).

Our final fMRI study sample included two groups, L_A_L_A_ (high-expressing “Long” genotype) and SS/SL_G_/L_G_L_G_ (low-expressing “Short” genotypes). The Long group (*N* = 21; 52% Male; Age: *M* = 20.8, *SD* = 8.6) did not differ from the Short group (*N* = 21; 48% Male; Age: *M* = 19.6, *SD* = 1.7) in age (*p* > 0.5), gender (*p* > 0.7) or ethnicity (*p* > 0.2). The Short group comprised low-expressing alleles were composed of individuals with the SS (*n* = 16), SL_G_ (*n* = 3), and L_G_L_G_ (*n* = 2) phenotypes.

### Stimulus materials

**Figure 1:**
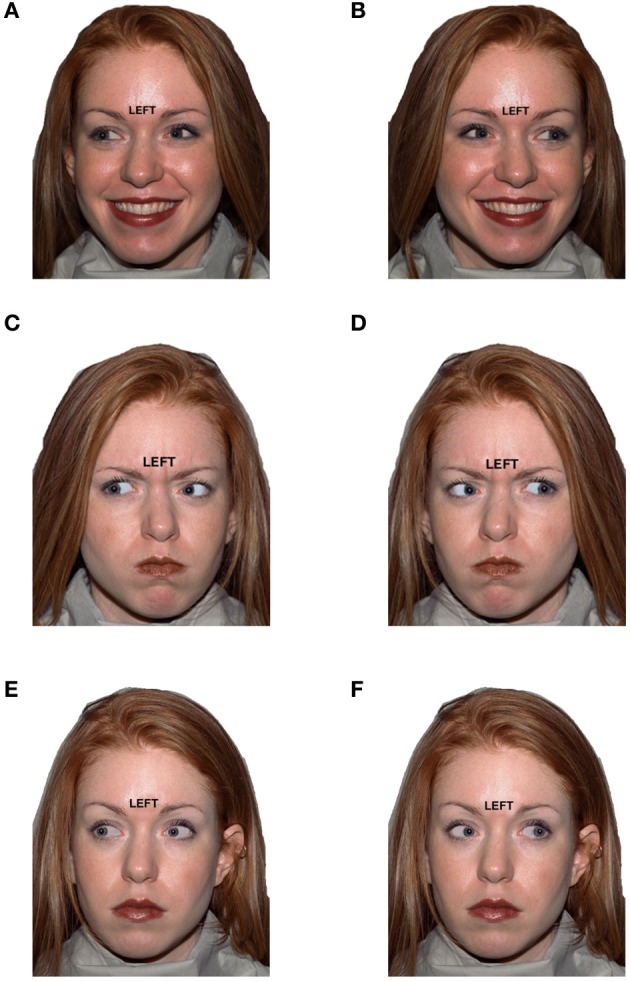
**Example stimuli for six conditions that varied by target direction to eye gaze (distractor) congruency and by emotional expression valence: (A) Happy/Congruent, (B) Happy/Incongruent, (C) Angry/Congruent, (D) Angry/Incongruent, (E) Neutral/Congruent, (F) Neutral/Incongruent**.

Stimuli consisted of faces selected from the NimStim stimuli (Tottenham et al., 2009) with a target direction (“LEFT” or “RIGHT”) printed just above the naison of face. The eye gaze, which was manipulated using Photoshop (Adobe, version CS2 software), could either be to the left or right (Figure 1). In addition, the emotional expression of the face was happy, angry, or neutral. Hence, the three key stimulus features were (1) target direction (task-relevant), (2) eye gaze (task-irrelevant) and (3) emotional expression (task-irrelevant). For congruent trials, target direction matched eye gaze (*LEFT-left* or *RIGHT-right*). For incongruent trials, target direction conflicted with eye gaze (*LEFT-right* or *RIGHT-left*). For conflict-neutral trials, eye gaze was straight ahead, and therefore neither conflicted nor matched the target direction word (*LEFT-straight ahead* or *RIGHT-straight ahead*). Thus, trials varied by target-gaze congruency (congruent, incongruent, conflict-neutral) and valence of emotional expression (Negative, Positive, Neutral), creating nine conditions: Negative Congruent, Negative Incongruent, Negative Conflict-Neutral, Positive Congruent, Positive Incongruent, Positive Conflict-Neutral, Neutral Congruent, Neutral Incongruent, and Neutral Conflict-Neutral (Figure 1). Conditions were equated for gender and other irrelevant stimulus features (e.g., hair color), as each condition contained the same 12 exemplar faces (6 male, 6 female).

### Procedure

All potential MRI participants were recruited between 2 and 8 months prior to scanning (Mean = 3.7 months, *SD* = 1.9), which did not differ across genotypes (*p* > 0.2). During an initial visit to the laboratory, all 221 participants completed the Neuroticism Extraversion Openness Five-Factor Inventory [(NEO-FFI Costa and McCrae, 1992)], and two computerized tasks designed to measure cognitive control, an N-back task (Stollstorff et al., 2010) and a Stop Signal Reaction Time Task (Logan et al., 1984), and provided a saliva sample for subsequent genotyping.

A subset of participants were invited to return for fMRI scanning based on their 5-HTTLPR homozygosity. On the day of scanning, they first received verbal instructions for the task outside the magnet, followed by an anatomical scan and the experimental task while undergoing fMRI scanning; they then completed the state anxiety questionnaire from the State-Trait Anxiety Inventory [STAI; (Spielberger and Vagg, 1984)] outside the magnet.

### Tasks and questionnaires performed outside the magnet

#### Trait negative and positive affect questionnaires

To obtain measures of negative and positive trait affect, which are suggested to be associated with the short and long 5-HTTLPR genotypes, respectively, we administered two questionnaires. The STAI is a self-report measure of state and trait anxiety that includes 20 statements, rated on a scale of 1 (not at all) to 4 (very much so), about the participant's immediate state of anxiety, and 20 statements, on a scale of 1 (almost never) to 4 (almost always), about trait anxiety. We used the overall percentile score derived from the STAI-State subscale (taken at the time of scanning) as a proxy for a trait tendency toward negative affect. The NEO is a questionnaire designed to measure a number of basic personality measures. We used the positive affect and negative affect subscales of the extraversion and neuroticism measures derived from the NEO as a proxy for a trait tendency toward positive and negative affect, respectively. The NEO-FFI was administered 2 and 8 months prior to scanning during the participants' initial visit to the laboratory; test-retest reliability for the NEO-FFI is quite high; 0.83 at 6 months (Murray et al., 2003).

#### Cognitive control tasks

To determine whether the two genotype groups varied in terms of basic cognitive control ability, we administered a variety of behavioral tasks designed to measure different aspects of cognitive control.

***N-back working memory***. This task was designed to measure aspects of cognitive control related to the ability to filter and update information in working memory. Participants completed a verbal *N*-back task, consisting of 6 alternating 1.2-min blocks of 1-, 2- and 3-back conditions (“low,” “medium,” and “high” working memory load, respectively). Each block comprised 24 trials preceded by an instruction screen stating the type of trial in the block (“1-back,” “2-back,” or “3-back”). For all conditions, one letter was presented on the screen at a time (for 0.5 s followed by a 2.5 s inter-trial interval) and the participant was instructed to press a button with their right index finger on the keyboard when the letter on the screen was the same as the one presented *n* trials previously. In the 1-back condition, participants were instructed to press the button if the letter was the same as the letter before it (e.g., “T” then “T”). In the 2-back condition, participants were instructed to press the button if the letter was the same as 2 before it (e.g., “R” then “L” then “R”); in the 3-back condition, participants were instructed to press the button if the letter was the same as 3 before it (e.g., “M” then “K” then “P” then “M”). The number of target responses was identical across trial conditions. Stimuli comprised consonants only; vowels were omitted to prevent encoding series of letters as pronounceable strings.

***SSRT (stop signal reaction time) task***. This task was administered to assess the ability to exert cognitive control to interrupt prepotent responses. Participants were instructed to press a button in response to a cue (an arrow pointing Left or Right) unless they saw a stop signal (a white square) presented immediately after the cue, in which case they were to withhold a button press on that trial. Each trial began with a visual masking stimulus presented for 200 ms, followed by a fixation ring. The fixation ring persisted for 200 ms, and was then followed by a left- or right-pointing arrow subtending approximately 2° of visual angle. Subjects were required to press the “z” key to left-pointing arrows, and the “m” key to right-pointing arrows as quickly and accurately as possible. On 25% of trials, these arrow stimuli were replaced by a white square after a variable “signal delay,” and subjects were required to inhibit their response to these stop signals. The signal delay was initially set to 250 ms and thereafter adjusted using an adaptive algorithm, such that the ISI was increased by 50ms following unsuccessful stop trials and decreased by 50ms following successful stop trials. SSRT was then calculated using the integration method, and was therefore equal to the nth percentile of Go signal RT minus the average SSD, where n corresponds to the proportion of successfully inhibited trials.

### Genotyping

Participants delivered 2 mL of saliva into a sterile 15 mL tube, after which the experimenter placed a cotton-tipped swab containing a lysis buffer consisting of 1% sodium dodecyl sulfate, TRIS buffer, and proteinase K. Tubes were delivered to the laboratory where the DNA was isolated using standard procedures, which were subsequently analyzed for 5-HTTLPR using a two-step process. First, the long (L) and short (S) variants were determined. The repeat polymorphism in the promoter region of the 5-HTT gene was genotyped by PCR as previously described (Lesch et al., 1996) using the following primers at concentrations of 10 μM; Forward: 5′- GGCGTTGCCGCTCTGAATGC -3′ Reverse: 5′-GAGGGACTGAGCTG-GACAACCAC-3′. PCR was performed using the AccuPrime™ GC-Rich DNA polymerase system (Invitrogen) with the following PCR program: 95°C for 10 min, followed by 35 cycles of 95°C for 30 s, 65°C for 30 s, and 72°C for 1 min. A final extension time of 72°C for 10 min was performed after the 35 cycles were complete. The PCR products were then run out on a 2% agarose gel stained with ethidium bromide. The amplification yielded distinct bands at 484 bp (S allele = 14 copies of repeat) and 528 bp (L allele = 16 copies of repeat), which were distinguished by a 100 bp DNA ladder run on the same gel. Second, the L_A_ and L_G_ variants were determined for the rs25531 single nucleotide polymorphism (SNP), present only on the long allele. Genotyping for rs25531 was performed by digesting the PCR products generated from the 5-HTTLPR PCR reactions with the restriction enzyme MspI (New England BioLabs). Specifically, 10 μL restriction digestion reactions were performed by combining 8 μL of the 5-HTTLPR PCR product, 1 μL of 10X NEBuffer 4, and 1 μL of MspI (concentration = 100,000 U/mL) and incubating the reactions for 2 h at 37°C followed by heat inactivation of the enzyme at 80°C for 20 min. The substitution of the G for A in the SNP produces an additional MspI recognition site (CCGG) on the long allele of the 5-HTTLPR PCR product. Genotypes were determined by running the digested PCR products out on a 2% agarose gel stained with ethidium bromide. Samples with two copies of the A allele for rs25531 showed a band at 340 bp (as well as bands at 127 and 62 bp due to multiple MspI recognition sites on the 5-HTTLPR PCR product), while samples with two copies of the G allele for rs25531 had additional digestion of the 340 bp product, yielding bands at 166 and 174 bp (as well as bands at 127 and 62 bp). Samples that were heterozygous for rs25531 showed a combination of these two band patterns.

### Imaging procedure

Imaging data were acquired using a 3T Siemens magnet (Siemens Magnetom Trio, Erlangen, Germany). Head movement was minimized by foam padding that held the subject's head in the coil firmly and comfortably. Prior to functional imaging, a high resolution sagittal T_1_-weighted structural scan was acquired using a 3D MPRAGE sequence with the following parameters: *TR* = 2530 ms, *TI* = 1200 ms, 256 × 256 mm FOV, 192-mm slab with 1-mm-thick slices, 256 × 256 × 192 matrix (effective resolution of 1.0 mm^3^), and a 7^o^ flip angle.

Participants viewed the stimuli via a mirror mounted on the coil that reflected the images that were projected onto a screen (209 × 279 cm) at the back of the bore of the magnet approximately 950 mm from the mirror. Stimuli were generated in E-prime (Version 2.0, Psychology Software Tools Inc., 2010) and viewed via a magnet-compatible projector. Fifty axial slices (3.4 × 3.4 × 4.0 mm) were positioned to be parallel to the base of orbitofrontal cortex and covering the whole brain (*TR* = 2500 ms, *TE* = 29 ms, 220 × 220 mm FOV, 75° flip angle). A total of 404 volume images were acquired over a single run (16:55 min) using a T2^*^-sensitive gradient EPI sequence.

Alternating task and fixation blocks were presented in counterbalanced order (same for each participant). Each task block comprised three out of nine experimental conditions; each block consisted of 10 trials. Each 2.5 s trial began with a face stimulus, which remained on the screen for 1 s. The face cleared and a fixation-cross appeared for 1.5 s. Participants could respond at any point during the trial to indicate the direction of the word on the forehead by pressing one of two buttons on a button box (with the right hand); the left button with Index finger for “LEFT” and the right button with middle finger for “RIGHT.” No feedback was provided. Fixation blocks consisted of five trials of a blank white screen (1 s) followed by a fixation cross (1.5 s), to which participants were instructed not to respond.

### fMRI processing and data analysis

Images were analyzed in SPM5 (www.fil.ion.ucl.ac.uk/spm). The first 4 volumes were discarded to allow for T1 equilibration effects, leaving 400 volumes. Images were corrected for slice acquisition timing and were then corrected for translational and rotational motion by realigning to the first image of the run. All subjects demonstrated less than 2 mm of absolute translational motion in any one direction and less than 2° of rotation around any one axis in each run. Images were coregistered with the high-resolution structural images of the participant. The structural images were segmented into separate gray and white matter images, and the gray matter image was normalized into standard MNI space by comparison with a template gray matter image. The normalization parameters used were then applied to the functional images to bring them into MNI space. All images were smoothed using a Gaussian kernel with full-width at half-maximum (FWHM) of 8 mm.

fMRI responses were modeled by a canonical hemodynamic response function. At the individual subject level, activation maps were generated using linear contrasts identifying regions that were more active during incongruent relative to congruent blocks (“interference/conflict contrast”), separately for each emotional valence condition.

Five second-level analyses were performed: (1) To identify clusters engaged by the Stroop-like task in general, a one-sample *t*-test on the conflict contrast was performed (all subjects and all valences). (2) To test whether emotionally neutral cognitive control activation did not differ between genotype groups, a 2-sample *t*-test was performed on the conflict contrast in the neutral-valence condition only. (3) To test our hypothesis of a 5-HTTLPR × Valence interaction, our key analysis of interest, a 2 × 2 mixed analysis of variance (ANOVA) with 5-HTTLPR (Long, Short) as a between-subject factor and Valence (Happy, Angry) as a within-subject factor was performed. For each analysis, maps were thresholded at *p* < 0.005, *k* = 150 which is an overall significance level of *p* < 0.05 corrected for multiple comparisons based on Monte Carlo simulation of random noise distribution [using 3dClustSim module of AFNI (Forman et al., 1995)]. To further examine the ANOVA, contrast estimates were extracted from activated clusters using MARSBAR (Brett et al., 2002) and analyzed for genotype and valence differences with *t*-tests. (4) To test which regions correlate with trait negative affect while viewing angry faces, for each genotype group separately, we ran a covariate analysis on the Incongruency Contrast (incongruent—congruent) for the negative valence (angry faces) condition only using the covariate of STAI state anxiety. (5) To test which regions correlate with trait positive affect while viewing happy faces, for each genotype group separately, we ran a covariate analysis on the Incongruency Contrast for the positive valence (happy faces) condition only using the covariate of scores on Positive Affect subscale of the NEO-FFI.

## Results

### Negative and positive affect

#### Self-report measures

A between-subjects ANOVA of subscales from the NEO-FFI revealed that mean Extraversion-Positive Affect scores were higher in Long (*M* = 16.48, *SD* = 2.01) than Short (*M* = 14.33, *SD* = 2.65) participants [*F*_(1, 41)_ = 8.69, *p* = 0.005, η^2^ = 0.82] and that Neuroticism-Negative Affect scores were marginally higher in Short (*M* = 14.67, *SD* = 2.09) than Long (*M* = 12.95, *SD* = 3.21) participants [*F*_(1, 41)_ = 3.29, *p* = 0.077, η^2^ = 0.42]. No other scales or subscales from the NEO-FFI were significant (*p*s > 0.1; Table 1 reports Extraversion and Neuroticism scales and subscales). A between-subjects ANOVA showed that mean percentile State anxiety scores from the STAI were higher in Short (*M* = 46.65, *SD* = 19.68) than Long (*M* = 33.95, *SD* = 20.04) participants [*F*_(1, 40)_ = 4.19, *p* = 0.048, η^2^ = 0.51]. Thus, the Short group scored higher on measures of Negative Affect as would be expected. In addition, the Long group scored higher on a measure of Positive Affect (see Table 1).

**Table 1:** **Demographics, cognitive control, and trait affect measures for short and long 5-HTTLPR genotype groups; mean (SD)**.

		**Short (SS/SL_G_/L_G_L_G_)**	**Long (L_A_L_A_)**	***p*-value**
**DEMOGRAPHICS**				
*N* (sample size)		21	21	1.0
Age in years		19.6 (1.7)	20.8 (8.6)	0.57
Gender		F: 11	F: 10	0.76
		M: 10	M: 11	
Ethnicity (No. of Caucasian)		18	21	0.18
**COGNITIVE CONTROL TASKS**				
***N*-back working memory**				
Accuracy	1-back:	95.9% (9)	96% (15)	0.98
	2-back:	95.5% (11)	92.3% (10)	0.34
	3-back:	81.6% (19)	84.8% (18)	0.61
Reaction Time	1-back:	597 ms (159)	556 ms (167)	0.45
	2-back:	674 ms (164)	678 ms (186)	0.95
	3-back:	747 ms (228)	723 ms (311)	0.79
**Stop signal reaction time (SSRT)**				
		220 ms (29)	222 ms (49)	0.90
**TRAIT AFFECT SELF-REPORT MEASURES**				
***STAI state anxiety***		46.6 (19)	33.9 (20)	0.048^*^
*Percentile score*				
*NEO-FFI*				
**Neuroticism**		30.05 (7)	27.74 (6)	0.19
Negative affect		14.67 (3)	12.95 (3)	0.07
Self-reproach		15.38 (5)	14.29 (5)	0.46
**Extraversion**		42.52 (5)	45.48 (8)	0.17
Positive affect		14.33 (3)	16.48 (2)	0.005^*^
Sociability		13.86 (2)	14.33 (4)	0.60
Activity		14.05 (3)	14.76 (3)	0.43

* Significant group difference.

### Cognitive control measures

To test whether groups were equivalent in cognitive control ability, we used two tasks that tap aspects of cognitive control: (1) the N-back task, designed to measure the ability to update and remove information from working memory; and (2) the Stop-Signal Reaction Time (SSRT) task, designed to measure inhibitory control over motoric responding.

#### N-back working memory

Groups did not differ in performance at any working memory load for accuracy (*p*s > 0.3) or reaction time (*p*s > 0.4), indicating that short and long genotype groups had similar working memory ability (Table 1).

#### SSRT

Groups did not differ in stop signal reaction time (*p* > 0.9), indicating that short and long genotype groups had similar inhibitory control ability (Table 1).

### Behavioral results

A response was scored as “correct” if the participant pressed the button (left or right) in accordance with the target direction, and “incorrect” if the opposite button was pressed or if there was no response within 1.5 s (“timed-out”; *M* = 0.002% of trials, which did not differ by genotype, *p* > 0.3). For each participant, mean accuracy (% correct) and mean reaction time (ms) for correct responses was computed for congruent and incongruent trials for each emotional valence (Table 1) and this was subsequently entered into 2 mixed 2 × 2 × 3 ANOVAs (for accuracy and reaction time, separately), with genotype (Short, Long) as a between-subjects factor and congruency (congruent, incongruent) and valence (happy, angry, neutral) as within-subject factors.

#### Accuracy

A main effect of congruency [*F*_(1, 40)_ = 15.66, *p* < 0.001, η^2^ = 0.28] indicated that participants were more accurate for congruent (*M* = 98.5%, *SD* = 2.3) than incongruent (*M* = 96.6%, *SD* = 4.8) trials. Thus, participants' accuracy exhibited an interference, or “Stroop” effect. No other main effects or interactions reached significance (*p*s > 0.1, see Table 2).

**Table 2:** **Mean accuracy (SD in parenthesis) and reaction time (in ms; SD in parentheses) for congruent and incongruent trials by emotional valence condition in short and long genotype carriers**.

			**Short *N* = 21**	**Long *N* = 21**
Accuracy	Angry	*Congruent*	98.4% (2.5)	98.8% (1.9)
		*Incongruent*	96.4% (5.0)	94.8% (4.9)
	Happy	*Congruent*	99.0% (1.8)	98.2% (2.1)
		*Incongruent*	97.0% (4.6)	98.0% (3.1)
	Neutral	*Congruent*	98.2% (2.5)	98.4% (3.1)
		*Incongruent*	97.2% (4.4)	96.4% (6.6)
Reaction Time	Angry	*Congruent*	569 (51)	585 (51)
		*Incongruent*	572 (51)	572 (48)
	Happy	*Congruent*	549 (56)	565 (61)
		*Incongruent*	576 (46)	595 (52)
	Neutral	*Congruent*	545 (55)	557 (66)
		*Incongruent*	562 (64)	568 (53)

#### Reaction time

A main effect of congruency [*F*_(1, 40)_ = 11.70, *p* < 0.001, η^2^ = 0.23] indicated that participants were faster to respond to congruent (*M* = 561 ms, *SD* = 56) than incongruent (*M* = 574 ms, *SD* = 52) trials. Thus, participants' response latencies exhibited an interference, or “Stroop” effect. There was a main effect of valence [*F*_(2, 80)_ = 8.43, *p* < 0.001, η^2^ = 0.17]; pairwise comparisons revealed that reaction time was significantly faster for the neutral emotion condition (*M* = 558 ms, *SD* = 59) than positive (*p* = 0.002; *M* = 571 ms, *SD* = 53) and negative (*p* = 0.001; *M* = 574 ms, *SD* = 50) emotional conditions, which did not differ from each other (*p* = 0.43). Furthermore, there was a congruency × valence interaction [*F*_(2, 80)_ = 11.47, *p* < 0.001, η^2^ = 0.22]; paired *t*-tests revealed that the interference effect (congruent faster than incongruent) was significant for neutral [*t*_(41)_ = 2.50, *p* = 0.016] and positive [*t*_(41)_ = 7.05, *p* < 0.001] valence conditions, but not for the negative valence condition [*t*_(41)_ = 0.80, *p* = 0.428]. Importantly, there was no main effect of genotype or interaction with genotype (*p*s > 0.3), indicating that the effect of congruency and valence did on reaction time did not differ by genotype (see Table 2).

### Neuroimaging results

#### Cognitive control activation—main effect of congruency

To ensure that our task engaged neural systems involved in cognitive control, we performed a one-sample *t*-test on the conflict contrast (incongruent > congruent) across all valences (i.e., all emotional expressions) for all participants. This analysis revealed activation in a wide-spread range of regions, most all of which are seen in tasks involving cognitive control (Table 3): right inferior and middle frontal gyri, right medial superior frontal gyrus, bilateral superior parietal gyrus/precuneus, right posterior superior temporal gyrus, right fusiform gyrus (fusiform face area; FFA) and left cerebellum.

**Table 3:** **Regions involved in negatively and positively valenced face processing (task minus fixation baseline contrast, *p* = 0.05 corrected)**.

	**BA**	**Voxels**	**Voxel coordinates**			***Z*-Score**
			***x***	***y***	***z***	
**MAIN EFFECT OF NEGATIVE EMOTION (ANGRY FACES > FIXATION)**						
Bilateral ventral visual stream		12220				
Right occipital (cuneus)	17/18		28	−94	6	24.18
Left occipital (cuneus)	17/18		−18	−102	4	23.50
Right fusiform face area (FFA)	37		40	−44	−20	18.50
Left fusiform face area (FFA)	37		−38	−48	−21	15.92
Right amygdala	n/a	364	20	−6	−16	6.19
Right putamen/ventral striatum			22	6	8	6.71
Left amygdala	n/a	1097	−16	−10	−12	6.83
Left putamen/ventral striatum			−22	2	8	6.85
Medial frontal gyrus/anterior cingulate	6	533	−6	8	52	10.94
Left middle frontal gyrus/premotor cortex	6	1430	−28	−2	48	6.88
Left superior parietal gyrus	7	908	−32	−60	50	6.45
**MAIN EFFECT OF POSITIVE EMOTION (HAPPY FACES > FIXATION)**						
Bilateral Ventral Visual Stream		11459				
Right occipital (cuneus)	17/18		26	−96	6	26.26
Left occipital (cuneus)	17/18		−20	−100	2	24.26
Right fusiform face area (FFA)	37		38	−48	−20	17.67
Left fusiform face area (FFA)	37		−40	−46	−20	14.68
Right amygdala	n/a	729	22	4	10	7.18
Right putamen/ventral striatum			28	6	−6	6.89
Left amygdala	n/a	925	−16	−8	−14	6.03
Left putamen/ventral striatum			−26	2	−8	8.15
Medial frontal gyrus/antierior cingulate	6	594	−6	6	54	10.93
Left middle frontal gyrus/premotor cortex	6	1068	−44	0	30	6.88
Right middle frontal gyrus/premotor cortex	6	576	44	6	54	5.96
Right middle frontal gyrus	46		46	30	38	5.62
Left superior parietal gyrus	7	1019	−28	−56	48	8.02
Right superior parietal gyrus	7	616	34	−56	48	6.56
**MAIN EFFECT OF VALENCE**						
*Negative > positive*						
Right fusiform face area (FFA)	37	271	42	−40	−18	3.83
Right posterior middle temporal gyrus	39		−50	−72	8	4.22
Left middle temporal gyrus	37/39	175	−54	−66	10	3.61
Right occipital	17/18	539	4	−86	−2	4.21
Left occipital			−12	−84	−6	3.54
*Positive > negative*						
Left posterior superior temporal sulcus (STS)	41	154	−40	−36	16	3.35

#### Effects of valence

To determine whether the faces were engaging emotional processing as we had hypothesized, we ran a number of contrasts. First, we examined the contrast of Faces with Negative Emotion vs. Fixation as well as the contrast of Faces with Positive Emotion vs. Fixation. These two contrasts revealed similar patterns, with extensive activation in the ventral visual processing stream, ventral striatum, and amygdala bilaterally (see Table 3, top). These latter findings indicate that our face stimuli did indeed engage regions involved in emotional processing. In addition, we compared activation for stimuli in which the face had a negative emotion compared to a positive one, which yielded great activation in visual cortex and portions of the superior temporal sulcus for negative compared to positive emotional expressions (see Table 3, bottom).

#### Group comparison of cognitive control activation—neutral emotion

Next we examined whether there were any differences in activation of cognitive control regions for the two genotype groups when there was no salient emotional expression of the face (i.e., the neutral facial expression). A 2-sample *t*-test (for the interference contrast, incongruent > congruent) for the neutral valenced (non-emotional) condition revealed that the Short group had more activation of left middle frontal gyrus and left posterior middle temporal gyrus relative to the Long group. The reverse comparison (Long > Short) revealed no significant group differences in activation (Table 3). This finding suggests that the short group may engage cognitive control regions more than the long group, but to a somewhat limited degree.

#### 5-HTTLPR × valence interaction

**Figure 2:**
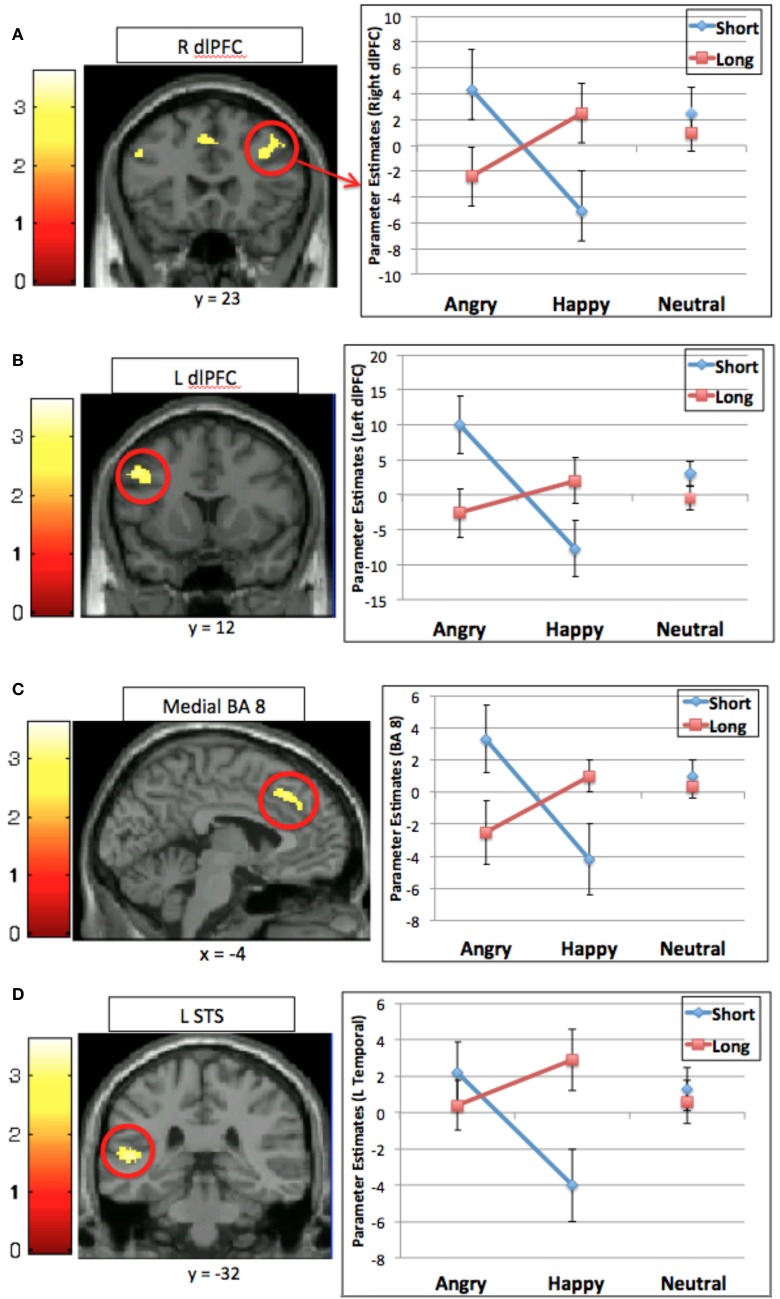
**Interaction between emotional valence and 5-HTTLPR for the interference contrast (incongruent > congruent) in four regions: (A) right dorsal lateral prefrontal cortex (R dlPFC); (B) left dorsal lateral prefrontal cortex (L dlPFC); (C) medial superior prefrontal cortex (BA 8); (D) left superior temporal sulcus (L STS).** Graphs show mean contrast estimates (± standard error) in the activated cluster by genotype and emotional valence.

To address the main question of interest, that is, whether genotype influences the degree to which neural systems involved in cognitive control are differentially engaged depending on the emotional nature of distracting stimuli, we performed a analysis to determine those brain regions that would exhibit a genotype × valence interaction for the interference contrast (incongruent > congruent trials). A significant effect was observed in four regions: bilateral middle prefrontal cortex, left medial superior PFC, and left posterior superior temporal gyrus (Table 3, Figure 2). Comparison of contrast estimates from each region revealed a similar pattern; that is, activation was higher in Short carriers relative to Long carriers for negatively-valenced faces, and higher in Long relative to Short carriers for positively-valenced faces (see Figure 2).

#### Individual differences analysis—fMRI

**Figure 3:**
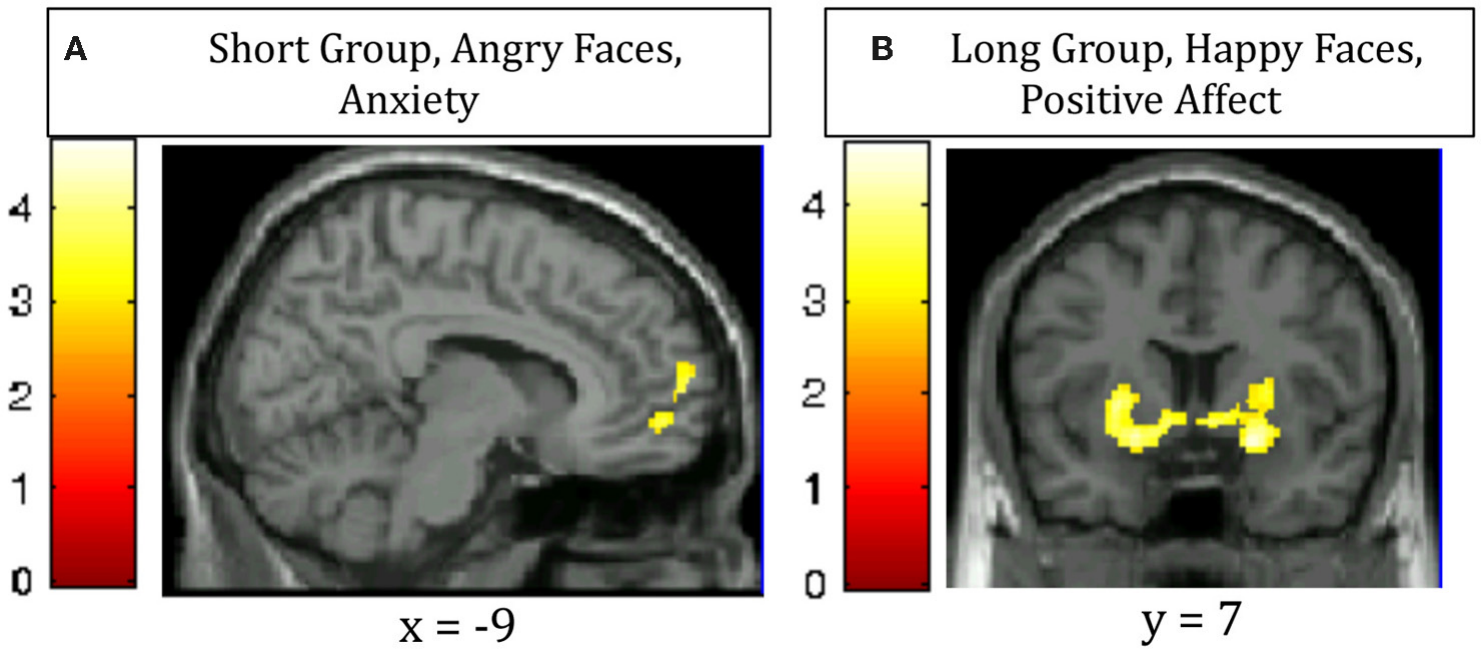
**Regions in which increased activation for the contrast of incongruent—congruent trials correlates with (A) increased anxiety in Short 5-HTTLPR individuals viewing Angry faces (ventromedial prefrontal cortex and frontal pole); (B) increased trait positive affect in Long 5-HTTLPR individuals viewing Happy faces (bilateral ventral striatum)**.

A covariate analysis using the interference contrast (incongruent minus congruent) was run for the negative valence condition (negative faces) using STAI state anxiety as the covariate in order to determine regions that are sensitive to cognitive conflict in a negative emotional context that vary by anxiety self-report in each group. This analysis in the Short group revealed that increased activation of the ventromedial prefrontal cortex and the frontal pole was associated with greater anxiety. The Long group did not show this pattern (Table 4, Figure 3). A similar covariate analysis using the interference contrast was run using the Negative Affect subscale from the NEO-Neuroticism questionnaire (assessed during initial visit 2–8 months prior to scanning). This analysis in the Short group while viewing angry faces revealed ventromedial prefrontal cortex, frontal pole, left middle frontal gyrus and left posterior middle temporal gyrus. The Long group did not show any significant activation (Table 4). A second complementary covariate analysis on the interference contrast was run for the positive valence condition (happy faces) using NEO-Positive Affect as the covariate in order to determine regions that are sensitive to cognitive conflict in a positive emotional context. In the Long group, greater activation in ventral striatum was associated with greater positive affect. The Short group did not show a similar pattern (Table 4, Figure 3).

**Table 4:** **Regions involved in cognitive control under various emotional conditions in individual carriers of the short and long 5-HTTLPR genotype (*p* = 0.05 corrected)**.

	**BA**	**Voxels**	**Voxel coordinates**			***Z*-Score**
			***x***	***y***	***z***	
**MAIN EFFECT OF CONGRUENCY (INCONGRUENT > CONGRUENT)**						
Right inferior prefrontal gyrus	45	186	52	22	−4	3.09
Right middle frontal gyrus	6/8/9	367	46	8	52	3.84
Right superior medial prefrontal gyrus	6/8	290	0	12	56	3.68
Right posterior superior temporal gyrus	21/22	626	62	−44	12	3.44
Right fusiform gyrus (FFA)	n/a	558	38	−50	−16	4.21
Right intraparietal sulcus/precuneus	7/40	964	30	−46	44	3.62
Left intraparietal sulcus/precuneus	7/40	241	−26	−52	44	3.20
Left cerebellum	n/a	233	−40	−70	−26	3.85
	n/a	706	−8	−76	−24	3.61
**NON−EMOTIONAL CONGRUENCY EFFECT (NEUTRAL FACES)**						
*Short > Long*						
Left middle frontal gyrus	6	184	−38	−4	40	3.52
Left posterior middle temporal gyrus	36	317	−54	−54	4	3.39
*Long > Short*	No significant clusters					
**GENOTYPE × VALENCE INTERACTION (**2** × **2** ANOVA)**						
Right middle prefrontal gyrus	9/46	201	48	34	26	3.07
Left middle/inferior prefrontal gyrus	9	408	−42	6	34	3.48
Medial superior prefrontal gyrus	8	218	−6	28	42	3.06
Left posterior superior/middle temporal gyrus	21/22	211	−50	−34	8	3.31
**TRAIT AFFECT COVARIATE ANALYSIS**						
*Short group, angry faces, anxiety*						
Frontal pole	10	166	14	62	6	3.75
Ventromedial prefrontal cortex	10/47		12	54	−6	3.20
*Long group, happy faces, positive affect*						
Left ventral striatum	n/a	1072	22	10	−14	3.74
Right ventral striatum	n/a		−20	8	−12	3.60
*Short group, angry faces, negative affect*						
Right frontal pole	10	107	12	54	20	3.39
Left frontal pole	10	73	−18	58	16	3.10
Ventromedial prefrontal cortex	11/47	78	−4	42	−14	3.14
Left middle frontal gyrus	9	212	−32	14	34	3.22
Left posterior middle temporal gyrus	21	152	−60	−52	−4	3.47

## Discussion

The present study clearly demonstrates an interaction between neural systems involved in cognitive control and those involved in emotional processing that varies with genotype. Our results demonstrate that the distracting effect of valenced emotional information, which engages the need for cognitive control, differs depending on an individual's allelles for the serotonin transporter genotype (5-HTTLPR). Specifically, when the distracting information was negatively-valenced, individuals carrying the Short genotype recruited prefrontal cognitive control regions to a greater extent than individuals with the Long genotype. In contrast, when the distracting emotional information was positively-valenced, individuals with the Long genotype recruited these regions to a greater extent than those with the Short genotype. Of note, these data do not simply show that one genotype has more activity in one region or one condition. Rather, this double-dissociation highlights the opposing effects depending on emotional valence and 5-HTTLPR genotype.

We interpret this finding as indicating that regions involved in cognitive control become engaged when emotional information is distracting in nature. What is distracting, however, depends, in part, on genotype. Supporting the idea that the valence of emotional information has differential affects depending on genotype was the pattern of activation in regions processing the emotional expression of the face, including the superior temporal sulcus. The Short genotype group exhibited greater activation for the negatively-valenced (i.e., angry) faces than the Long genotype group and the Long genotype group exhibited greater activation for the positively-valenced happy faces than the Short genotype group.

Two findings regarding our groups and their phenotypes are important. First, our behavioral data (in addition to the pattern of activation in regions processing facial expression discussed above), suggest differential processing of emotional information. The sample of individuals selected as homozygous for the short serotonin-transporter (5-HTTLPR) genotype had higher self-reported negative affect, while the long serotonin-transporter genotype had higher self-reported positive affect. Of note, these results suggest, moreover, that our sample is relatively representative, as this pattern is consistent with previous findings.

Second, in contrast to the clear group differences in the processing of emotional information, we found little evidence for group differences in their ability to exert cognitive control generally. We included assessment of cognitive control ability on two standard behavioral measures, the N-back task and the Stop-Signal Reaction Time Task, which tap different aspects of executive function. The former assesses the ability to manipulate the contents of working memory while the latter assessed the ability to override a pre-potent response. The groups performed equivalently. Obviously, one cannot draw strong conclusions from a null result as it may reflect a Type 1 error. However, the pattern of differences in emotional self-report combined with no differences on tasks of cognitive control, supports the possibility that genotype is mainly influencing the processing of emotional information.

Also supporting this speculation are the neuroimaging results for faces with a neutral emotional expression. This analysis revealed only minor group differences in activation, which were observed in the left posterior middle temporal gyrus and the middle frontal gyrus with increased activation for the Short group. This finding is consistent with the idea that there are not large differences between the groups in the basic ability to engage neural mechanisms involved in cognitive control. Rather, such a pattern suggests that any differences in activation of cognitive control regions are more influenced by bottom-up effects, with increased sensitivity to the neutral facial expression in the short than long group (as evidenced by the activity in the left posterior middle temporal gyrus), which then, in turn, engages cognitive control. We speculate that for the short group, a neutral facial expression may not really be perceived as neutral, but potentially somewhat negatively valenced (Bistricky et al., 2011). Although other studies have found reductions in activation in prefrontal regions involved in cognitive control in individuals with depressive tendencies (Herrington et al., 2010) individuals in those studies have more severe trait negative affect. Our short carriers, however, did not have such high levels of negative affect, probably accounting for the relative lack of group differences in activation of prefrontal regions involved in cognitive control.

Rather than group differences in activation of cognitive control regions in general, the engagement of cognitive control regions in our task appears to be driven by the interaction of genotype and emotional valence. Aside from regions of the posterior superior temporal gyrus, which likely reflect group differences in processing of facial expression, all remaining regions showing a significant interaction of genotype and valence are involved in cognitive control. More specifically, the genotype by valence interaction was observed for activations in regions of the middle prefrontal cortex bilaterally, extending from the inferior frontal junction toward anterior portions of BA 9 and medial BA 8 in the cingulate gyrus extending upwards into pre-SMA. These are regions implicated across a large number of studies as playing an important role in cognitive control.

We postulate that the prefrontal regions (bilateral IFJ and medial pre-SMA), which are consistently active in paradigms requiring cognitive control such as the Stroop task (Nee et al., 2007), are engaged differentially by emotional valence due to differing cognitive control demands experienced by each group based on 5-HTTLPR status. These bottom-up differences in sensitivity to affective information, despite the fact such information is peripheral to the task and therefore task-irrelevant, nonetheless place additional demands on cognitive control, as such affective information is likely to capture attention. Cognitive control of Short genotype carriers is heightened when there is distracting emotional information of a negative nature, while that of Long carriers is heightened when there is distracting emotional information of a positive nature. We propose that this affective attentional bias feeds forward to trigger cognitive control to suppress task-irrelevant information (eye-gaze for emotional facial expressions) and increase attention toward task-relevant information. This attentional interference then gives rise to differential engagement of prefrontal regions. Moreover, we speculate that such top-down control is sufficient to control bottom-up affective biases so as to not influence behavior, as we found no significant differences in performance as a function of genotype, measured either by accuracy or reaction time. Of course, we cannot preclude the possibility that the lack of differences in behavioral performance reflect other mechanisms besides compensatory activation of brain regions involved in top-down control.

Our research expands upon existing findings in a number of ways. While prior neuroimaging studies have demonstrated differential neural responses in attentional biases to emotional information based on the serotonin transporter genotype (Pérez-Edgar et al., 2010) and behavioral studies have shown that groups differ in cognitive control ability depending on emotional valence (Koizumi et al., 2010), our study is the first to show differential engagement of neural systems for cognitive control over such emotional biases based on serotonin transporter genotype. We also show that these attentional biases influence engagement of cognitive control not only for the 5-HTTLPR Short carriers, but also for the 5-HTTLPR Long carriers. Typically, the negative consequences of the 5-HTTLPR genotype is associated with the short allele (e.g., increase risk of affective disorder and negative personality traits). However, in our paradigm we show that a bias toward processing task-irrelevant positive information (in the Long group) can engage the need for activation of regions involved in cognitive control just as much as a bias toward processing task-irrelevant negative information (in the Short group). This highlights the extra cognitive burden for Long carriers in positive contexts, a potential downside to this allele typically associated with “positive” outcomes (see discussion by Homberg and Lesch, 2011).

Our correlational analyses revealed individual variation within each group as well. While viewing angry faces, Short carriers who had higher anxiety tended to have higher activation of the ventromedial prefrontal cortex (vmPFC) and frontal polar regions, known to be involved in affective modulation and reappraisal (Diekhof et al., 2011). In a similar analysis, Short carriers who reported higher negative affect in their initial visit 2–8 months prior to scanning also tended to have higher activation of these regions (vmPFC and frontal pole) while viewing angry faces. While viewing happy faces, Long carriers who had higher positive affect tended to have more activation of the ventral striatum, known to be involved in reward processing (Haber and Knutson, 2010). These correlations were not present in control analyses (e.g., in Short carriers, positive affect did not correlate with any brain region). Thus, Short carriers who seem to have more extreme negative bias recruit regions that could suppress the negative affect, while Long carriers who seem to have high positive affect engage the reward system when “in their element” (i.e., happy faces promoting a positive context).

Although the present results are intriguing, a limitation of the present study is its small sample size (*N* = 42). Thus, replication would be advisable. However, an advantage of the current study, relative to most other fMRI studies of this kind, is that we included only homozygotes. Most fMRI studies of 5-HTTLPR differences include heterozygous carriers of both the Short and Long alleles (S/La) into one or the other group (S/S or La/La), thereby diminishing possible group differences and possibly clouding analyses. Future studies will need to explore the phenotype, both behaviorally and with regards to neural activation, displayed by heterozygotes. In addition, our results do not clearly isolate the process that is affected by cognitive control, whether it be a reduction in bias toward certain types of emotional information, an increased ability to deal with conflict, either at the perceptual or response level, or some other process.

In sum, our results further our understanding of the neural mechanisms underlying the inherent emotional biases of homozygous 5-HTTLPR Short carriers as compared to the inherent emotional biases of homozygous 5-HTTLPR Long carriers. Both groups show heightened engagement of face processing regions, but do so differentially depending on the valence of the face. For the Short Group, greater activity is observed in these regions when the task-irrelevant facial expression is negative in valence. In contrast, for the Long group, greater activity is observed when the task-irrelevant facial expression is positive in valence. Increased activation, and likely attention, to such task-irrelevant information appears to engage cognitive control for both groups, but differentially depending on valence. Our work suggests that when assessing the interplay between emotion and cognition, consideration of genotype, in this case related to 5-HTTLPR status, may play an important role.

### Conflict of interest statement

The authors declare that the research was conducted in the absence of any commercial or financial relationships that could be construed as a potential conflict of interest.
